# VITADIAL “Does correction of 25 OH-VITAmin D with cholecalciferol supplementation increase muscle strength in hemoDIALysis patients?”: study protocol for a randomized controlled trial

**DOI:** 10.1186/s13063-021-05302-9

**Published:** 2021-05-25

**Authors:** Stanislas Bataille, Nathalie Pedinielli, Elisa Carreno, Mathilde Prezelin-Reydit, Philippe Chauveau, Guillaume Jean, Thomas Robert, Mickaël Bobot, Guillaume Seret, Elisabeth Jouve, Frederic Lavainne, Marianne Serveaux, Laurence Vrigneaud, Stéphanie Gentile

**Affiliations:** 1 Phocean Institute of Nephrology, Marseille, France; 2 ELSAN, Clinique Bouchard, Marseille, France; 3 Aix-Marseille Univ, C2VN, INSERM, INRAE, Marseille, France; 4 AURAD Aquitaine, Gradignan, France; 5 NephroCare Tassin-Charcot, Ste Foy-Lès-Lyon, France; 6 Centre de Néphrologie et Transplantation Rénale, Hôpital de la Conception, AP-HM, Marseille, France; 7 Association ECHO, Pôle Santé Sud, Le Mans, France; 8 Aix Marseille Univ, School of medicine - La Timone Medical Campus, EA 3279: CEReSS - Health Service Research and Quality of life Center, Marseille, France; 9 Association ECHO, Polyclinique de l’Atlantique, Saint Herblain, France; 10 ATUP, Marseille, France; 11 Hôpital privé La Louvière, Ramsay Santé, Lille, France

## Abstract

**Background:**

Muscle strength decreases as kidney failure progresses. Low muscle strength affects more than 50% of hemodialysis patients and leads to daily life activities impairment. In the general population, numerous studies have linked low 25OH-vitamin D (25OHD) concentrations to the loss of the muscle strength and low physical performances. Data on native vitamin D and muscle function are scarce in the chronic kidney disease (CKD) population, but low 25OHD levels have been associated with poor muscle strength. We present in this article the protocol of an ongoing study named VITADIAL testing if cholecalciferol supplementation in hemodialysis patients with low 25OHD improves their muscle strength.

**Methods/design:**

VITADIAL is a prospective open randomized French multicenter study. All patients will have 25OHD levels ≤50nmol/L at randomization. One group will receive 100,000 UI cholecalciferol once a month during 6 months; the other group will receive no treatment during 6 months.

In order to randomize patients with 25OHD ≤50nmol/L, supplemented patients will undergo a 3 months wash-out period renewable 3 times (maximum of 12 months wash-out) until 25OHD reaches a level ≤50nmol/L.

The main objective of this study is to analyze if a 6-month period of oral cholecalciferol (i.e., native vitamin D) supplementation improves muscle strength of hemodialysis patients with low 25OHD vitamin D levels. Muscle strength will be assessed at 0, 3, and 6 months, by handgrip strength measured with a quantitative dynamometer.

Secondary objectives are (1) to analyze 25OHD plasma levels after vitamin D wash-out and/or supplementation, as well as factors associated with 25OHD lowering speed during wash-out, and (2) to analyze if this supplementation improves patient’s autonomy, reduces frailty risk, and improves quality of life.

Fifty-four patients are needed in each group to meet our main objective.

**Discussion:**

In the general population, around 30 randomized studies analyzed the effects of vitamin D supplementation on muscle strength. These studies had very different designs, sizes, and studied population. Globally, these studies and the meta-analysis of studies favor a beneficial effect of vitamin D supplementation on muscle strength, but this effect is mainly found in the subgroup of aged patients and those with the lowest 25OHD concentrations at inclusion.

We reported a positive independent association between 25OHD and handgrip strength in a population of 130 hemodialysis patients in a dose-dependent manner. In our cohort, a plateau effect was observed above 75 nmol/L. Only two randomized studies analyzed the effect of native vitamin D supplementation on muscle strength in hemodialysis patients, but unfortunately, these two studies were underpowered. VITADIAL is a trial specifically designed to assess whether cholecalciferol might benefit to hemodialysis patient’s muscle strength.

**Trial registration:**

ClinicalTrials.govNCT04262934. Registered on 10 February 2020 - Retrospectively registered.

## Background

### Muscle strength in hemodialysis patients

It has been known for 50 years that chronic kidney disease (CKD) patients have low muscle strength and low physical exercise capacities [1, 2]. Muscle strength decreases as kidney failure progresses. Low muscle strength affects more than 50% of hemodialysis patients and leads to impairment on activities of daily living [3–5].

Body composition is also modified in hemodialysis patients: fat mass is increased and muscle mass is decreased [5, 6]. Our team has reported previously that 88% of patients in a hemodialysis center had low muscle strength but only 33% low muscle mass, suggesting that muscle strength might be due to low muscle fibers functioning more than muscle atrophy [5]. Striated muscle histological changes have been reported during kidney failure, with myofibrosis, fat inclusions within normal muscle fibers called myosteatosis, and type 2 fibers atrophy [7, 8]. Rapid contraction of striated muscle is mainly due to type 2 fibers, where type 1 fibers are essential for standing. Type 2 fibers are crucial to avoid falls. Other morphological anomalies have also been described in kidney failure: a reduced number of capillaries per number of fibers, a low number of mitochondria, mitochondrial dysfunction [8, 9]. Electrophysiological anomalies in peripheral motor nerves of hemodialysis patients are slight, with mainly a reduction in muscle relaxation following type 2 fibers atrophy [10]. These alterations lead to a reduced maximum oxygen consumption (VO2 max) with low muscle endurance as well as low muscle strength [11].

Numerous other factors have been proposed to explain the loss of muscle strength in CKD patients: protein energy wasting syndrome, lack of exercise, comorbidities, such as diabetes or heart failure, treatments, or uremic toxins [9, 12–14]. Within these factors, vitamin D deficiency has been proposed [15].

### Vitamin D and vitamin D insufficiency in hemodialysis patients

Native vitamin D deficiency (25OHD level <50 nmol/L) or insufficiency (level between 50 and 75 nmol/L) is very frequent in chronic kidney disease patients, with more than 80% having low serum 25OHD levels [16, 17]. Some observational data report an increasing rate of vitamin D deficiency with worsening of kidney function where other studies report serum vitamin D levels comparable in patients with CKD and patients with normal kidney function [18, 19].

Many causes and risk factors have been associated with low 25OHD levels in hemodialysis patients: age, female gender, high adiposity, low physical activity, and diabetes mellitus [16]. Interestingly, metabolism of vitamin D is also modified in uremic patients in some specific ways: reduced vitamin D receptor (VDR) and increased CYP24A1 levels, impaired 25OHD tubular resorption, reduced skin synthesis of vitamin D [20, 21].

In CKD patients, native vitamin D is less transformed into active 1–25 vitamin D—namely calcitriol—because 1α-hydroxylation is mainly performed by the kidney, but almost every cell type is able to produce 1α-hydroxylase and to act in an autocrine or paracrine manner. Native vitamin D supplementation increases 25OHD levels, but also calcitriol levels in the CKD population [22, 23]. Moreover, although 25OHD has a low affinity for the vitamin D receptor (VDR), in the plasma, its concentration is at least 100 times higher than the calcitriol concentration. Therefore, native vitamin D is important as it might have different metabolic effects than calcitriol, including in skeletal muscle cells [22, 24].

### Effects of vitamin D on muscle strength in the general population

Vitamin D deficiency is common in the general western population, with at least 50% of the population affected according to the Workshop Consensus for Vitamin D Nutritional Guidelines [25]. This proportion reaches 80% of the French adult population according to the recent epidemiological French studies [26]. Beyond the classical effects on bone metabolism, vitamin D regulates numerous physiological effects. Vitamin D receptor is expressed among almost all cellular types including muscle cells [27]. In vitro, 25OHD is implicated in myoblast cell proliferation in culture. In rats, vitamin D injection is implicated in the recruitment, proliferation, and differentiation of satellite cells into mature muscle fibers and is essential for wounding repair [28]. Vitamin D is not only necessary for muscle repair, but is also required for mature muscle fiber contraction and mitochondrial metabolism [29].

In the general population, numerous observational studies have linked low 25OHD concentrations to the loss of the muscle strength and low physical performances, to a higher incidence of falls, to muscle pain, and to myosteatosis [30–37]. These observational studies led to the realization of around 30 randomized studies analyzing the effects of vitamin D supplementation on muscle strength. These studies had different designs, sizes and non-homogenous studied populations [38]. Moreover, their results regarding vitamin D are sometimes inconclusive because of the different type**s** of vitamin D supplementation or the association with calcium supplements [39]. Globally, these studies and the meta-analysis of studies favor a beneficial effect of vitamin D supplementation on muscle strength, but this effect is mainly found in the subgroup of the most aged patients and those with the lowest 25OHD concentrations at inclusion [38, 39]. To note, if vitamin D supplementation has any effect, it could be mainly observed in the population with vitamin D deficiency, i.e., with the lowest 25OHD [40]. This is an important clue since many studies regarding vitamin D supplementation have been performed in patients with normal or non-measured 25OHD concentrations, and fail to show any improvement of muscle strength [41, 42]. These latter results must not be extrapolated to the population with low 25OHD [39].

Regarding the risk of falls, a first meta-analysis published in 2004 including 1237 participants concluded that vitamin D supplementation reduced risk of falling by 22% (corrected OR, 0.78; 95% confidence interval [CI], 0.64–0.92) compared to calcium or placebo [43]. This study included only 5 studies. Since then, a larger meta-analysis which included 37 randomized trials (*n*=34144) did not find any effect analysis of supplementation on falls, but this meta-analysis included heterogeneous studies, many of them did not report baseline 25OHD concentrations or had normal baseline 25OHD [44].

Could vitamin D supplementation be deleterious for muscle function? This question has been raised by an Australian study in 2256 women aged over 70 years old who received 500,000 UI cholecalciferol supplementation yearly and in which the incidence of falls was higher in the group of patients with supplementation [45]. The design of this study was criticized because it led to high 25OHD concentrations. Nevertheless, two other studies have raised concerns on high doses and the risk of femoral neck fractures or falls [46, 47].

### Effects of vitamin D on muscle strength in the hemodialysis population

Data on native vitamin D and muscle function are scarce in the CKD population, and most interventional studies have been performed with calcitriol or vitamin D analogs such as paricalcitol [48–55].

Few studies reported an association between low 25OHD and low muscle strength in hemodialysis patients [56]. In 25 hemodialysis patients, Boudville et al. reported a positive correlation between 25OHD levels and quadriceps strength, even if most patients were taking calcitriol therapy [24]. We reported a positive independent association between 25OHD and handgrip strength in a population of 130 hemodialysis patients in a dose-dependent manner [15]. Interestingly, in our cohort, a plateau effect was observed above 75 nmol/L.

Only two randomized studies analyzed the effect of native vitamin D supplementation on muscle strength, but unfortunately, these two studies are underpowered. Marckman et al. randomized 52 CKD patients of which 27 hemodialysis patients with 25OHD < 50nmol/L to an 8-week long treatment of 40,000 UI cholecalciferol per week or placebo. Cholecalciferol had no effect on muscle function (secondary objective) in this small study [57]. In the second study, Hewitt et al. included 60 hemodialysis patients with 25OHD < 60nmol/L to 50,000 UI cholecalciferol or placebo, once per week during 8 weeks and then once per month during 4 months. This study failed to show any difference in muscle strength of upper and lower limb or functional performances between supplemented and not supplemented patients [58].

So far, no study has been published that had calculated the number of patients to include in order to show an effect of native vitamin D on muscle strength in hemodialysis patients. For this reason, we set up the VITADIAL study. VITADIAL is a prospective randomized trial designed to assess whether cholecalciferol supplementation improves hemodialysis patient’s muscle strength.

## Methods

### Study design and setting

**Fig. 1:**
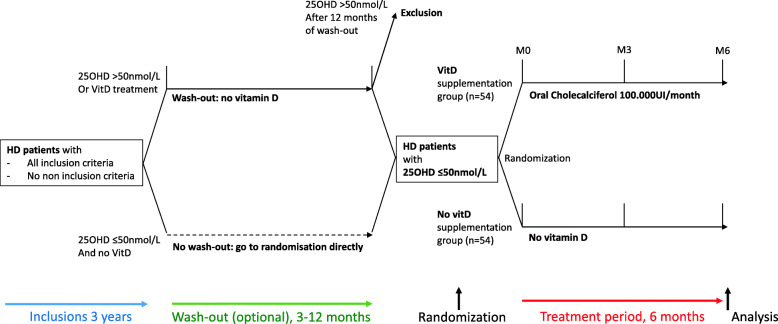
Study design. 25OHD: 25-hydoxyvitamin D; VitD: Vitamin D

VITADIAL is a prospective open randomized French multicenter study. Serum 25OHD levels will be ≤ 50 nmol/L at randomization. Vitamin D group will receive 100,000 UI cholecalciferol once a month during 6 months; control group will receive no treatment during 6 months (Fig. 1).

In France, hemodialysis patients with low 25OHD are often supplemented with cholecalciferol. Thus, to randomize patients with 25OHD ≤ 50nmol/L, supplemented patients will undergo a 3 months wash-out period renewable 3 times (maximum of 12 months wash-out) until serum 25OHD reaches a level ≤ 50nmol/L.

Patients with 25OHD ≤ 50nmol/L at inclusion will directly be randomized. Patients with 25OHD > 50nmol/L at inclusion will undergo wash-out and be randomized as soon as 25OHD is ≤ 50nmol/L. Patients with 25OHD still > 50nmol/L after 12 months of wash-out will be excluded of the study (Fig. 1).

### Objectives and judgment criteria

The main objective of this study is to analyze if a 6-month period of oral cholecalciferol (i.e., native vitamin D) supplementation improves muscle strength of hemodialysis patients with low 25OHD vitamin D levels. Muscle strength will be assessed by handgrip strength measured with a quantitative dynamometer (Kern, Germany). Measures will be performed on both arms before the hemodialysis session in a standardized procedure. Handgrip strength measurement is a well validated evaluation of muscle strength in the hemodialysis population [15, 59, 60].

Secondary objectives are (1) to analyze 25OHD plasma levels after vitamin D wash-out and/or supplementation, as well as factors associated with 25OHD lowering speed during wash-out, and (2) to analyze if this supplementation improves patient’s autonomy, reduces frailty risk, and improves quality of life. Patient autonomy will be measured by the Activities of Daily Living-Katz index which is a standardized measure of biological and psychosocial function [61]. Frailty risk will be measured by the auto questionnaire Frail-Non-Disabled FIND which is validated scoring method of frailty risk [62]. Quality of life will be measured by the Kidney Disease Quality Of Life scoring method (KDQoL-SF™) [63].

### Study intervention

Study protocol is described in Table 1. All patients of participating dialysis centers will be screened for inclusion and non-inclusion criteria. If every inclusion criterion is met, but no non-inclusion criterion, investigators will inform patients and collect their written informed consent.

**Table 1:** Study procedures

	Screening	Inclusion	Wash-out phase	Treatment/no treatment (random phase)		
	V1	V2	V3 and/or V3bis and/or V3ter and/or V3quater	V4/M0	V5/M3	V6/M6
Check of selection criteria	✓					
Patient information	✓	✓				
Check of inclusion criteria		✓				
Consent Form Signature		✓				
Socio-demographic data		✓				
Clinical data		✓	✓	✓	✓	✓
Biological data		✓	✓	✓	✓	✓
Including 25OHD, iPTH, calcemia, phosphor		✓	✓	✓	✓	✓
Randomization				✓		
Dispensation treatment/no treatment				✓	✓	✓
Main criterion: handgrip		✓	✓	✓	✓	✓
Secondary criteria: ADL, FIND, KDQOL scores				✓	✓	✓
Compliance			✓	✓	✓	✓

During the wash-out period, all vitamin D supplementation (native or active) will be suspended. All other medical treatments and dialysis prescriptions will be maintained as usual care. 25OHD will be measured every 3 months as well as handgrip strength. If 25OHD lowers or equals to 50 nmol/L, the patient will be randomized. If not, the patient will continue wash-out for 3 additional months. After 12 months of washout, if 25OHD remains > 50 nmol/L, the patient will be excluded.

After randomization, muscle strength will be measured at baseline (at randomization) and 3 and 6 months. Variation of muscle strength at randomization and at the end of the study will be compared in the two groups.

During the treatment period, patients randomized in the cholecalciferol group will receive 100,000 UI oral cholecalciferol every month during 6 months, but no other vitamin D supplementation; patients randomized in the control group will receive no vitamin D at all. Medication will be provided to dialysis facilities; it will be given by the investigator at the end of dialysis session in the dialysis facility and the medication will be traced by the investigator. All other medical treatments and dialysis prescriptions will be maintained as usual care. Muscle strength will be measured at randomization, 3 and 6 months. Secondary judgment criteria will be measured at randomization and the end of the study. After 6 months with or without cholecalciferol supplementation, the study will end.

During the whole study, intact parathyroid hormone (iPTH), calcium and phosphate will also be measured every 3 months to screen for adverse events.

### Study population and sample size

CKD Patients on hemodialysis for more than 3 months, aged over 18 years old, who gave their consent will be included (Table 2).

**Table 2:** Inclusion, non-inclusion, and drop-out criteria

Inclusion criteria: - Under hemodialysis for more than 3 months - Aged over 18 years old - Gave their consent	
Non-inclusion criteria: - Non-fluent French speaker - Incapacity to provide consent or to answer questionnaires - Pregnancy or breast feeding - Cognitive impairment - Bedridden or life expectancy <1 year - Active cancer - Uncontrolled hyperparathyroidism as defined by the K-DIGO (iPTH >9x normal laboratory maximal value), cinacalcet treatment or hypocalcemia <2.0 mmol/L or hypercalcemia >2.7mmol/L - Past osteoporosis fracture - Treatment with active vitamin D - Unable to perform handgrip measurement - 25OHD >50nmol/L without vitamin D treatment - Cholecalciferol intolerance or allergy	
Drop out criteria: - 25OHD >50nmol/L after 12 months wash-out - Hypercalcemia >2.7mmol/L - Hyperparathyroidism (iPTH >9x normal laboratory maximal value) during wash-out or after randomization if patient is in the no treatment group - Hypoparathyroidism (iPTH <3x normal laboratory lower value) in a patient receiving cholecalciferol - Cholecalciferol intolerance or allergy - Death, renal transplantation - Pregnancy - Consent withdrawal - Renal recuperation allowing hemodialysis to stop - Unability to perform handgrip	

Inclusion, non-inclusion and drop-out criteria are described in Table 2. Hyperparathyroidism was defined as the Kidney Disease Improving Global Outcome (KDIGO) by an iPTH level >9 times the upper normal limit of the assay [64].

Sample size was calculated in order to meet our main objective and based on an observational study showing a dose-dependent correlation between 25OHD and muscle strength measured by handgrip [15]. In this study, mean muscle strength was 13 ± 8 kg in patients with 25OHD < 50 nmol/L and 18 ± 8 kg in patients with 25OHD > 75 nmol/L. To show a 5-kg difference between the two groups, considering a standard deviation of 8 kg, an α-risk of 0.05, and a power of 0.9, the number of patients required is 54 patients in each group (*t* test comparison of 2 independent means, https://marne.u707.jussieu.fr/biostatgv/). The study will continue until 110 patients will reach the end of the study according to the protocol.

In order to achieve recruitment, all hemodialysis patients from participating centers will be screened for eligibility every three months (concomitant to PTH and vitamin D dosages). The study will be proposed to all patients with inclusion criteria and no non-inclusion criteria. If recruitment in participating centers is not sufficient, more centers should be included in the study.

### Biological dosing

In order to facilitate patient recruitment and retention, biological analysis will be performed in the local usual hemodialysis center laboratories. As each center might have different dosing techniques, randomization will be stratified by center. Labs were asked to inform if any dosing technique was to change during the study regarding calcium, phosphate, iPTH, or 25OHD dosing.

Biological values will be performed every 3 months during the wash-out and treatment phase to search for drop-out criteria or the possibility to randomize patients.

### Randomization

Randomization will be centralized in the coordination center of the study, at Phocean Institute of Nephrology, Marseille, France. Patients will be randomly allocated to each arm (treatment or no treatment) in a 1:1 ratio between the two arms using a computerized random program (MINIM, program for randomizing patients to treatment groups in clinical trials by the method of minimization, by Stephen Evans, Simon Day and Patrick Royston) stratified per center, age (< or ≥ 70 years old) and gender. MINIM is a program for randomizing patients to treatment groups in clinical trials by the method of minimization. This software randomly assigns the first participants, then accounts for the covariates of participants (center, age, gender) previously enrolled, and assigns each new participant to the group that provides better balance.

### Statistical analysis

A flow-chart diagram will describe the number of patients by status (inclusion, drop-out, randomization, follow-up). Patient characteristics will be described by treatment groups: qualitative data will be expressed as number and percentages, and quantitative data as mean and 95% confidence interval. Comparison between the two groups will be performed using the Chi-2 test for qualitative variables and the Student test for quantitative variables. If conditions to use these tests are not met, comparisons will be performed using appropriate non-parametric tests (Fischer’s exact test, Mann-Whitney test). Analyses will be considered as significant if *p* value is ≤ 0.05, in a bilateral situation.

The main objective will be analyzed by comparing muscle strength variation during the treatment period (M0–M6) between treated (receiving cholecalciferol) and non-treated group using a covariance analysis in which principal analyzed effect will be the treatment group and will be included as covariables the initial muscle strength, the study center, age stratification (< or ≥ 70 years old), and gender. Principal analysis will be based on intention to treat principle.

Secondary analyses will compare the ADL, FIND, and KDQOL scores between the two treatment groups using the same statistical method (analysis of covariance). The decreased of 25OHD plasma level will be described at the end of the wash-out period (mean change from inclusion and number of patients with 25OHD ≤ 50 nmol/l). To analyze factors associated with 25OHD lowering speed, a linear (or the most appropriate function) mixed model (random intercept and/or slope) will be performed including variables potentially related to 25OHD changes, such as but not limited to age, gender, BMI, diabetes, and duration of previous 25OHD treatment.

### Data monitoring

Data quality will be monitored by the clinical researcher at the end of the study. Data in the Case Report Form will be compared to source data for 10% of included patients. If the rate of inappropriate data exceeds 5%, data monitoring will be performed in all the data of the concerned research center.

### Protection of human subjects

All patients will provide written consent and will be informed on their right to withdraw from the study at any time. The study has been approved by an ethics committee (see the “Ethics approval and consent to participate” paragraph).

In case of protocol amendment, the promoter of the study will previously obtain the ethics committee and the healthcare authorities’ agreement. A new written consent will be obtained from previously included participants.

## Trial status

Protocol version number and date are V5.0 2020/06/02. The protocol recruitment began on 01/02/2018 and the recruitment will be completed by 01/01/2022. We aim to publish the results of this trial during year 2022.

## Discussion

VITADIAL is the first study designed with a sufficient size to analyze the impact of native vitamin D supplementation on muscle strength in hemodialysis patients. Many factors influence muscle strength in the specific population, thus, only a randomized study will be able to answer this important question. Unfortunately, the two studies published in literature assessing the effect of native vitamin D supplementation on muscle strength were less powered and shorter (4 months) which could have limited the chance to show any benefit [57, 58].

In France, practice in many hemodialysis centers is to give oral cholecalciferol monthly to hemodialysis patients exerting low 25OHD. To our knowledge, no randomized study has shown a clinical benefice of this practice. To note, this practice is common in France, but not in all other countries. Safety during the wash-out period or in the non-treatment arm will be closely monitored by calcium, phosphate, iPTH dosages, but we believe this period is at low clinical risk. On the other side, vitamin D intoxication occurs only with 25OHD concentrations are higher than 375 nmol/L [65]. In a French study by Jean et al. describing the evolution of 25OHD levels in a hemodialysis patient cohort after monthly 100,000 UI cholecalciferol supplementation similar to our protocol, serum 25OHD levels increase from 26.8 to 102 nmol/L after 6 months, which is far below the toxicity level, and no peak effect was observed after the first cholecalciferol dose [66].

The open design of our protocol should also be discussed. We agree that the higher level of proof studies is the double-blind designed. However, our aim was to reach a sufficient number of patients ending the study as expected by the protocol, we thus needed to facilitate the realization and participation to VITADIAL. Importantly, when setting up the study protocol, we did not have external funding and a placebo was too expensive. Nevertheless, our study will be the largest study exploring the effect of cholecalciferol on muscle strength in hemodialysis patients and we chose to favor a greater number of inclusions even if we had to accept an open-label design. To note, our primary endpoint is muscle strength which is an objective measurement and needs no investigator interpretation.

VITADIAL is also the only study assessing 25OHD concentrations variations after interrupting native vitamin D supplementation in the hemodialysis population. The rapid or low decrease of 25OHD concentrations after stopping supplementation could provide clues on vitamin D physiology in this condition (low or important organ storage).

In conclusion, VITADIAL is a French prospective open-labeled randomized study designed to analyze the effect of cholecalciferol on muscle strength in hemodialysis patients. Safety of this study as well as all ethics and regulatory authorities has been assessed. Our study is currently underway and will provide clues to the clinical benefits of vitamin D supplementation as well as vitamin D metabolism in this specific population of patients.

## Data Availability

Data and material will be available on justified demand at the Phocean Nephrology Institute, Clinique Bouchard, Marseille, France.
